# Predicting antifreeze proteins with weighted generalized dipeptide composition and multi-regression feature selection ensemble

**DOI:** 10.1186/s12859-021-04251-z

**Published:** 2021-06-23

**Authors:** Shunfang Wang, Lin Deng, Xinnan Xia, Zicheng Cao, Yu Fei

**Affiliations:** 1 Department of Computer Science and Engineering, School of Information Science and Engineering, Yunnan University, Kunming, 650504 China; 2 School of Public Health (Shenzhen), Sun Yat-Sen University, Guangzhou, 510006 China; 3 School of Statistics and Mathematics, Yunnan University of Finance and Economics, Kunming, 650221 China

**Keywords:** Antifreeze proteins prediction, Weighted general dipeptide composition, Lasso regression, Ridge regression, Ensemble feature selection, Two-stage multiple regressions

## Abstract

**Background:**

Antifreeze proteins (AFPs) are a group of proteins that inhibit body fluids from growing to ice crystals and thus improve biological antifreeze ability. It is vital to the survival of living organisms in extremely cold environments. However, little research is performed on sequences feature extraction and selection for antifreeze proteins classification in the structure and function prediction, which is of great significance.

**Results:**

In this paper, to predict the antifreeze proteins, a feature representation of weighted generalized dipeptide composition (W-GDipC) and an ensemble feature selection based on two-stage and multi-regression method (LRMR-Ri) are proposed. Specifically, four feature selection algorithms: Lasso regression, Ridge regression, Maximal information coefficient and Relief are used to select the feature sets, respectively, which is the first stage of LRMR-Ri method. If there exists a common feature subset among the above four sets, it is the optimal subset; otherwise we use Ridge regression to select the optimal subset from the public set pooled by the four sets, which is the second stage of LRMR-Ri. The LRMR-Ri method combined with W-GDipC was performed both on the antifreeze proteins dataset (binary classification), and on the membrane protein dataset (multiple classification). Experimental results show that this method has good performance in support vector machine (SVM), decision tree (DT) and stochastic gradient descent (SGD). The values of ACC, RE and MCC of LRMR-Ri and W-GDipC with antifreeze proteins dataset and SVM classifier have reached as high as 95.56%, 97.06% and 0.9105, respectively, much higher than those of each single method: Lasso, Ridge, Mic and Relief, nearly 13% higher than single Lasso for ACC.

**Conclusion:**

The experimental results show that the proposed LRMR-Ri and W-GDipC method can significantly improve the accuracy of antifreeze proteins prediction compared with other similar single feature methods. In addition, our method has also achieved good results in the classification and prediction of membrane proteins, which verifies its widely reliability to a certain extent.

## Background

Antifreeze proteins (AFPs) are proteins that inhibit ice recrystallization by reducing the freezing point of intracellular fluids. It enables fish, insects, plants, algae, bacteria and other organisms to survive in extremely cold environments [1]. It is essential for the survival of organisms in extremely cold environments [2].

In the 1950s, Scholander et al. [3] first observed that certain fish could survive at temperatures below their body fluid freezing point. Until 1997, some researchers first discovered that this antifreeze substance, which can make the living body tolerant of coldness, is a special protein and was named as antifreeze proteins [4]. In 2002, Davies et al. successively found that AFP can adsorb on the surface of ice crystals. The interaction between AFP and ice crystals has a significant effect on the overall growth of ice [5]. The identification of new AFPs plays an important role in understanding the interaction of proteins with ice and the creation of new ice-binding domains in other proteins. Because of the wide variety of antifreeze proteins, identifying new antifreeze proteins is a challenge for biologists. Identifying antifreeze proteins in organisms by traditional biotechnology is time-consuming and costly. With the rapid growth of sequences genomic data, dealing with large amounts of biological sequences data requires fast and accurate automated methods for identification and annotation. Therefore, many research groups are dedicated to the study of biological sequences extraction algorithms, feature selection, and classification algorithms using machine learning and deep learning methods, such as amino acid composition (AAC), pseudo amino acid composition (PseAAC), protein position-specific scoring matrix (PSSM), dipeptide composition (DipC), tripeptide composition (TPC), 20-D condensed feature vectors (CFV), general dipeptide composition (GDipC), Lasso feature selection, neural network (NN), support vector machine (SVM), k-nearest neighbor (KNN), random forest (RF) and decision tree (DT), etc., and successfully applied them to protein structure and functional spectrum classification and prediction [6–23]. Recently Stochastic Gradient Descent (SGD) has been successfully applied to the field of sparse and large-scale machine learning [24, 25]. Strictly, SGD is only an optimization technique, not a specific machine learning model. However, it is a both simple and efficient method to fit linear classifiers and regressors under convex loss functions such as squared loss.

Until 2011, Daswamy et al. applied machine learning technology to antifreeze proteins prediction for the first time. In their proposed method, a variety of physicochemical properties were used to encode the features of the protein sequences, and a random forest algorithm was used as the classifier [26]. This innovation has attracted wide attention of researchers to related fields, and many researchers have joined the antifreeze proteins sequences research. In 2012, Zhao et al. proposed a prediction method called AFP_PSSM, which used support vector machine (SVM) and position-specific scoring matrix (PSSM) to predict antifreeze protein. PSSM was applied to predict antifreeze proteins for the first time in this study [27]. In 2014, Mondal et al. proposed a method called AFP-PseAAC in which Chou's pseudo-amino acid composition (PseAAC) was successfully applied to the prediction of antifreeze proteins with an accuracy of 84.75% [28]. In 2015, based on previous research, Yang et al. proposed an AFP recognition system called AFP-Ensemble, in which random forest classifiers were trained by combining different training subsets. Then it was aggregated into a consensus classifier by majority vote, and finally, the experiment achieved good results [29]. In the same year, He et al. proposed a new AFP predictor based on antifreeze proteins sequences, called TargetFreeze, whose main idea is the weighted combination of multiple protein extraction methods, including amino acid composition (AAC), pseudo amino acid composition (PseAAC) and position-specific scoring matrix (PSSM), and used support vector machine as the classifier [30]. In 2016, Xiao et al. proposed a method called iAFP-Ense based on AFP-PseAAC, whose main idea is to combine the gray model and the PSSM expression with PseAAC, and then to integrate eleven different random forest classifiers through the voting system. Xiao’s final experimental results show that the performance of the predictor is better than AFP-PseAAC [31]. In 2017, Pratiwi et al. built a Web server for classifying antifreeze proteins from non-antifreeze proteins, and the predictor has good performance with an accuracy of 88.82%. In addition, the server annotated AFPS using statistical and principal component analysis propensity scores and important physical and chemical properties [32]. Recently, Khan et al. proposed a method called RAFP-Pred, which first used information gain (IG) to extract feature of antifreeze proteins sequences and classified them with random forests. Experimental results show that the method has better robustness [33]. To further improve the robustness of the predictive model, Nath et al. used k-means clustering algorithm to create diverse and balanced training and test set, overcoming the shortcomings of random segmentation, making the model more generalized and robustness [34].

At present, researchers usually combine multiple extraction methods of protein sequences to make the obtained protein sequences more comprehensive, but this will cause a lot of information redundancy. Feature selection methods can effectively solve information redundancy. Therefore, in this paper, we propose an improved feature extraction method for antifreeze proteins, called weighted generalized dipeptide composition (W-GDipC), and an ensemble feature selection method based on two-stage multiple regressions, called LRMR-Ri. In the study, we first discuss the weighted coefficients in the fusion expression and then perform the fusion feature expression on the support vector machine (SVM), decision tree (DT) and stochastic gradient descent (SGD) classification algorithms. The experiment was carried out and the effectiveness of the method was verified by a five-fold cross-validation based on five evaluation indicators, accuracy, recall, precision, F-Measure and Matheus correlation coefficient. Finally, four commonly used feature selection algorithms, Lasso, Ridge, Maximal information coefficient (Mic), and Relief are introduced to process high-dimensional protein data. To avoid the local optimal problem coming from each single algorithm of above four, and to remove the redundancy to a greater extent, the ensemble feature selection (LRMR-Ri) based on two-stage multiple regressions is proposed, which is carried out both on the antifreeze proteins dataset (binary-classification problem) and on the membrane protein dataset (multi-classification problem), respectively. From the experimental results and related analysis, it can be seen that the proposed W-GDipC and LRMR-Ri methods can obtain excellent prediction results.

## Results and discussion

### The selection result of fusion coefficient

**Fig. 1:**
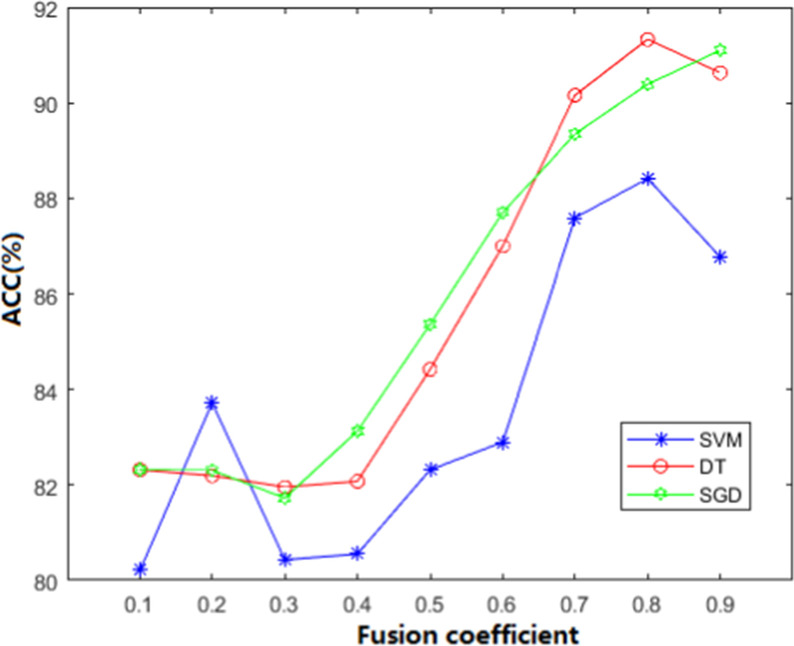
Accuracy of W-GDipC with different fusion coefficients under three classification algorithms for antifreeze proteins dataset

In order to reflect the effectiveness of W-GDipC and make the fusing effect optimal, we conducted several groups of comparative experiments with different weights. The threshold of fusion coefficient is set from 0 to 1, which is increased by 10% proportionately in each experiment. In order to show the influence of different weights of DipC and GDipC on the accuracy in the fusion process more clearly and intuitively, we plotted the change of the accuracy under different fusion coefficients based on three different classifiers SVM, DT, and SGD, as shown in Fig. 1.

It can be seen from Fig. 1 that with the increase of the fusion coefficient, the accuracy of three classifiers are all greatly improved, which shows that the features extracted by the DipC method are more recognizable. The higher the proportion of DipC is, the higher the recognition rate. When α is 0.8, the accuracy of DT is the highest among the nine groups of weight distribution experiments based on three different classification algorithms. At this time, the accuracy is 91.33% and the fusion expression of W-GDipC is (0.8DipC + 0.2GDipC). The accuracy was 2.92% and 0.23% higher than the highest values obtained by support vector machine (SVM) and stochastic gradient descent (SGD) at (0.8DipC + 0.2GDipC) and (0.9DipC + 0.1GDipC), respectively. Therefore, when the fusion coefficient is 0.8, the weight proportion of feature vectors extracted by DipC about 80%. At this time, the overall prediction effect is the best.

### Comparison of sequences feature extraction algorithms

In order to further evaluate the effectiveness of the proposed W-GDipC feature extraction, two single feature expression methods, GDipC and DipC, are used to compare with W-GDipC. The SVM, DT and SGD classifiers with different features were constructed by five-fold cross-validation for comparison. The average accuracy and Matthew correlation coefficient after the five-fold cross-validation were obtained.

**Fig. 2:**
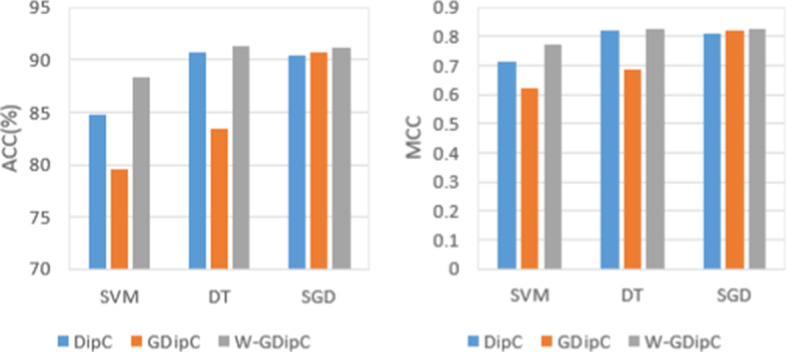
Evaluation of three feature extraction methods for antifreeze proteins dataset

From the experimental results, no matter which classifier is used, the ACC of W-GDipC is better than those of GDipC and DipC. Similarly, MCC of W-GDipC is better than those of GDipC and DipC. Specifically, in the prediction results with SVM, the average accuracy of W-GDipC, GDipC and DipC are 88.41%, 79.51% and 84.78%, respectively, and the average MCCs are 0.7704, 0.6232 and 0.7161, respectively. For classifier DT, the average accuracy of W-GDipC, GDipC and DipC are 91.33%, 83.37% and 90.75% respectively, and the average MCCs are 0.8254, 0.6854 and 0.8188 respectively. With classification algorithm SGD, the average accuracy of W-GDipC, GDipC and DipC are 91.10%, 90.76% and 90.40% respectively, and the average MCCs are 0.8239, 0.8232 and 0.8093, respectively. These data are sufficient to demonstrate the effectiveness of W-GDipC in the feature representation of antifreeze proteins sequences. In order to more intuitively see the prediction effects of three features representation under different classification algorithms, we drew the following Fig. 2.

It can be seen from Fig. 2 that the cross-validation results of W-GDipC in the three classification algorithms are better than the two single feature representation methods. This is because the length of the antifreeze proteins sequences is mostly in the range of 50–100, while GDipC is more suitable for short peptide and specific functionally prominent biological sequences, which are mainly used to enrich partial sequences features. And the features extracted by DipC are more likely to lead to local data sparsity. The W-GDipC proposed in this paper can optimize the feature representation of the two feature extraction methods on the antifreeze proteins sequences to a certain extent, because it contains more features than the former two, so that the evaluation index has a robust improvement compared with the first two feature extraction algorithms.

### Comparison of sequences feature selection methods

In order to verify the effectiveness of the proposed method, based on the feature representation of W-GDipC, the above antifreeze proteins dataset was still used as the benchmark dataset 1, and the classification models constructed by LRMR-Ri and the original feature selection algorithms based on DT, SVM and SGD classification algorithms is verified by five-fold cross-validation respectively. The values of evaluation indexes are given in Table 1.

**Table 1:** Evaluation based on four singer filters and LRMR-Ri for antifreeze proteins dataset with three models

Model	Evaluating indicator	Lasso	Ridge	Mic	Relief	LRMR-Ri
DT	RE (%)	77.65	90.59	88.82	92.94	95.88
	ACC (%)	76.93	92.39	89.81	91.34	93.21
	MCC	0.5328	0.8545	0.7932	0.8283	0.8627
SVM	RE (%)	77.65	95.88	87.06	94.71	97.06
	ACC (%)	82.79	93.56	90.75	93.21	95.56
	MCC	0.6574	0.9001	0.8138	0.8639	0.9105
SGD	RE (%)	76.47	88.82	81.87	87.13	91.76
	ACC (%)	81.87	85.38	83.04	87.13	90.53
	MCC	0.6675	0.7205	0.6698	0.7450	0.8332

According to the Table 1, for the antifreeze proteins dataset, the conclusions of the three models DT, SVM and SGD are generally consistent, that is, the values of RE, ACC and MCC of LRMR-Ri are all much larger than those corresponding values of single filters Lasso, Ridge, Mic and Relief. Among the four single filters (feature selection methods), it is easily seen that the performance of Lasso is the worst among the four, Mic is the third, the results of Ridge and Relief are close and both good while on the whole Ridge is better than Relief, which is the reason why Ridge is chosen in the second stage of LRMR-Ri method. Specifically, in the prediction model of DT, the differences of LRMR-Ri and Lasso are 18.23%, 16.28% and 0.3299 for RE, ACC and MCC, respectively, these differences are 19.41%, 12.77% and 0.2531 for SVM model and 15.29%, 8.66% and 0.1657 for SGD model, showing a generally outstanding performance of the proposed LRMR-Ri, for example, with classifier SVM, whose ACC value is nearly 13% higher than that of Lasso. The prediction effect obtained by the Lasso method is the worst compared with the other three single filters. This may be due to the excessive compression of non-zero coefficients in the Lasso analysis, which increases the deviation of the estimation results, resulting in poor prediction performance. And Lasso uses L1 regularization to make it easier to make part of the weights take 0, making the weights sparse; and Ridge uses L2 regularization can only make the weights close to 0, rarely equal to 0. This may also be the reason why the prediction effect of Ridge is higher than that of Lasso.

**Fig. 3:**
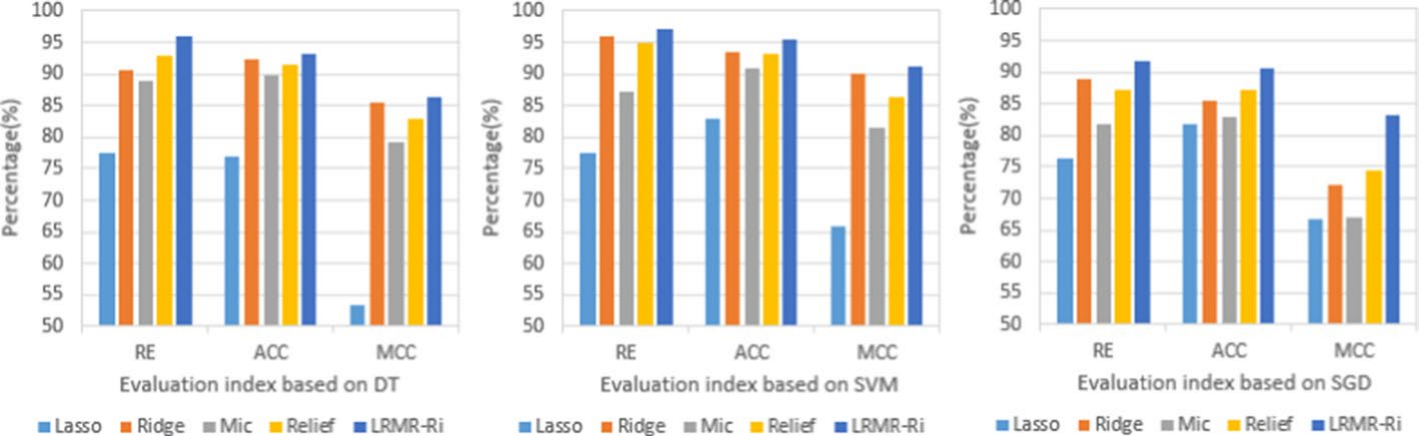
Evaluation of antifreeze proteins prediction by different feature selection methods based on three classification algorithms

Figure 3 provides a more intuitive view of the performance comparisons among the models. As can be seen from Fig. 3, the proposed LRMR-Ri method is superior to the other four methods, Lasso, Ridge, Mic and Relief for the prediction of antifreeze proteins. Therefore, it can be reasonably concluded that redundant features and local optimal or sub-optimal feature subsets are screened out to a great extent and a more effective feature subset is extracted by LRMR-Ri method.

### Prediction of membrane protein types by LRMR-Ri method

The prediction of these antifreeze proteins mentioned above is a binary classification problem, but membrane protein prediction belongs to the multi-classification problem. In order to verify the conclusion that the proposed method (and the fusion coefficient α of W-GdipC is still 0.8) is also effective to the multi- classification prediction, we carried out the related comparison experiment. The average PE, RE, F-Measure of eight categories and the overall ACC and MCC of membrane proteins are calculated based on different feature selection methods and different models, whose values are shown in Table 2.

**Table 2:** Evaluation based on four singer filters and LRMR-Ri for membrane protein dataset with three models

Model	Evaluating indicator	Lasso	Ridge	Mic	Relief	LRMR-Ri
DT	PE	0.3296	0.4928	0.3487	0.6778	0.4585
	RE	0.3350	0.3760	0.3425	0.4376	0.4258
	F-Measure	0.3316	0.4021	0.3443	0.4339	0.4353
	ACC (%)	69.86	77.97	71.82	77.59	79.83
	MCC	0.5037	0.6101	0.5036	0.6171	0.6563
SVM	PE	0.5446	0.5352	0.5228	0.5569	0.5159
	RE	0.4853	0.4391	0.4762	0.4574	0.4432
	F-Measure	0.5051	0.4616	0.4910	0.4842	0.4549
	ACC (%)	65.68	67.41	67.70	68.43	72.57
	MCC	0.5133	0.5766	0.5601	0.5951	0.6007
SGD	PE	0.3487	0.5777	0.6304	0.4776	0.5593
	RE	0.3425	0.4614	0.3911	0.3847	0.4460
	F-Measure	0.3443	0.4934	0.4384	0.4076	0.4810
	ACC (%)	71.82	78.19	75.88	75.88	80.15
	MCC	0.5237	0.6304	0.5648	0.5827	0.6562

In Table 2, the ACC of the proposed LRMR-Ri method can reach as high as 80.15% based on SGD algorithm, around 9% higher than of Lasso. As far as ACC and MCC values are concerned, the performance of LRMR-Ri on membrane protein dataset is still better than that of the other four feature extraction methods no matter with which model. For the indexes of PE, RE and F-Measure, the performance of LRMR-Ri is generally average.

**Fig. 4:**
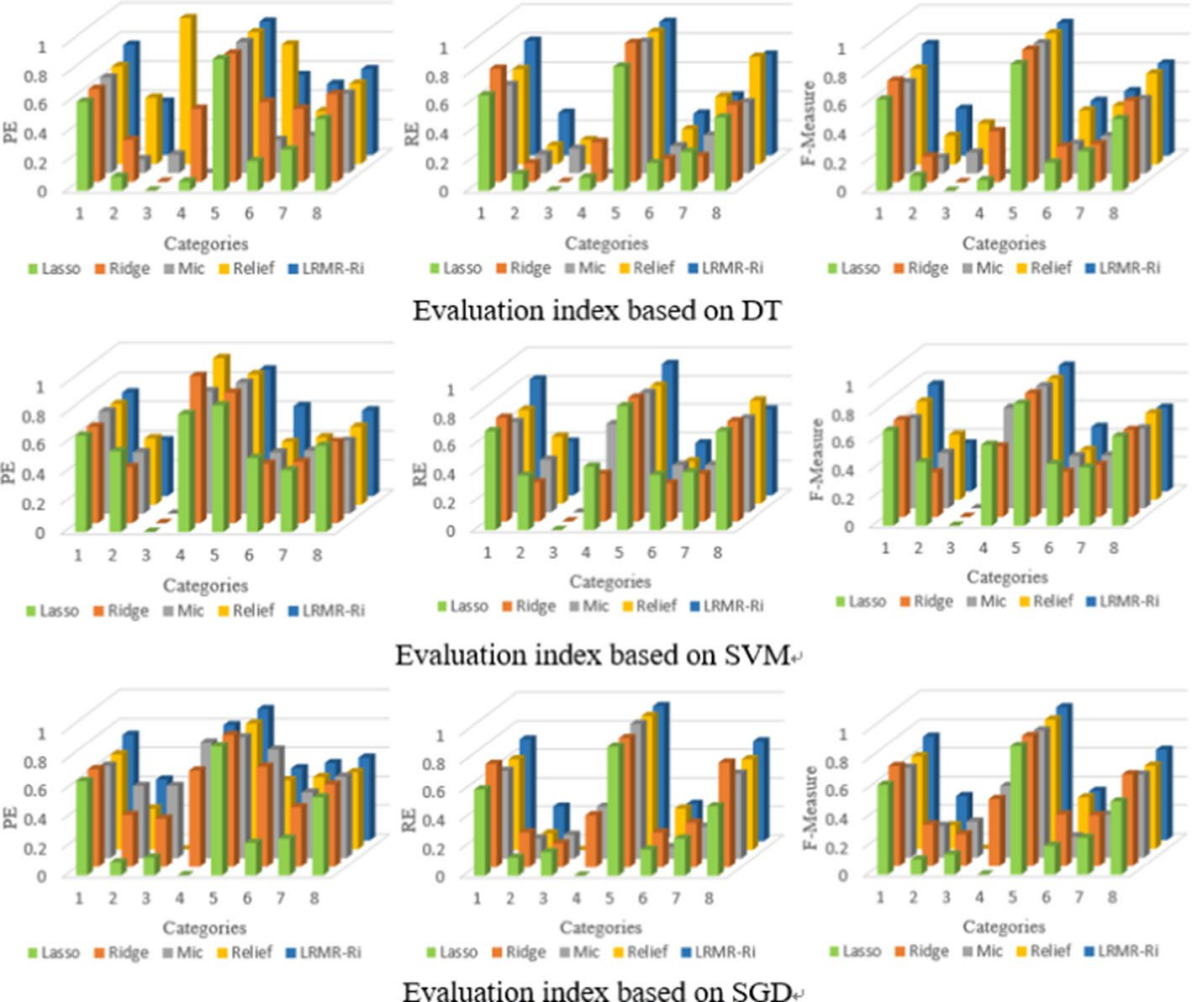
Prediction and evaluation of eight categories of membrane proteins by different feature selection methods and models

Figure 4 shows the evaluation of eight categories of membrane proteins by three different classification algorithms, DT, SVM and SGD with different indexes. No Three classification algorithms consistently suggested that, some indexes of categories 3 or 4 are 0, which intuitively reflects that the values of *TP* and *FP* of some categories are both zero in the process of prediction, indicating that those categories have not been recognized. This is due to the much too small number of samples in these two categories (only 30 sequences in category 3 and 56 sequences in category 4), whose training subsets are 24 and 44, respectively, resulting in poor learning results. On the contrary, the indicator values of category 5 (including 4581 sequences) are greater than 0.8 and higher than those of all other categories, showing that the more samples trained for the classification are, the better the prediction effect will be.

**Fig. 5:**
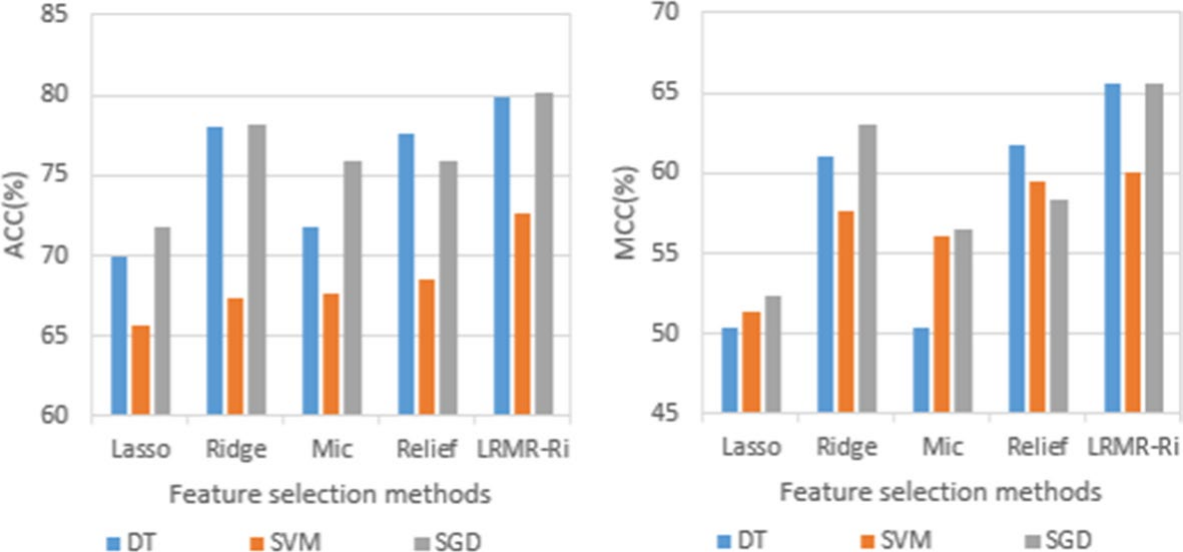
Membrane protein prediction with ACC and MCC by three classification algorithms and five feature selection methods

Figure 5 gives the overall prediction results of membrane protein types with ACC and MCC by three classification algorithms DT, SVM and SGD based on five feature selection methods. Even though there still exist 0 values of the predicted PE, RE or F-Measure for certain categories with small sample sizes, the overall accuracy and Matthew correlation coefficient of LRMR-Ri are relatively good among all, which verifies certain effectiveness of the proposed method in the multi-classification of membrane proteins.

### Comparison of antifreeze proteins with other machine learning methods

We compare the method proposed in this paper with the other three classifiers that also predict the antifreeze proteins by machine learning methods. The three methods are: AFP-Pred [26], AFP-PseAAC [28], TargetFreeze [30]. It can be seen from Table 3 that although the prediction performance of W-GDipC combined with multi-regression feature selection ensemble method using SVM as classifier is not good, but the prediction performance of SGD and DT as classifiers is better than other methods. And the prediction performance of DT as the classifier is the best, MCC and ACC are 0.158 and 7.9% higher than the AFP-Pred method, respectively. The poor prediction performance of using SVM as a classifier may be due to the small number of samples in the dataset we use. Comparison of antifreeze proteins with other machine learning methods shown in Table 3.

**Table 3:** Comparison of our proposed method with other methods for antifreeze proteins dataset

Method	MCC	ACC (%)
AFP-Pred	0.6674	83.38
AFP-PseAAC	0.800	89.69
TargetFreeze	0.819	90.95
W-GDipC + SVM	0.7704	88.41
W-GDipC + SGD	0.8239	91.10
W-GDipC + DT	0.8254	91.33

## Conclusion

In this work, we proposed a new feature representation method and a new feature selection method for predicting the antifreeze proteins, W- GDipC and LRMR-Ri. As far as W-GDipC, it uses a linear weighted fusion of GDipC and DipC. When the fusion coefficient is 0.8, the overall prediction effect is the best. The average accuracy of five-fold cross-validation of W-GDipC based on SVM, DT and SGD is 88.41%, 91.33% andis 88.41%, 91.33% and 91.10%, respectively. The average Matthew correlation coefficients are 0.7704, 0.8254 and 0.8239 respectively. The results show that the cross-validation results of W-GDipC in three classifiers are superior to two single feature expression methods. Then the LRMR-Ri method was used to select the features of high-dimensional antifreeze proteins. LRMR-Ri method constructs classification models based on DT, SVM, and SGD classification algorithms on antifreeze proteins dataset, and achieves ACC of 93.21%, 95.56% and 90.53% respectively in five-fold cross-validation. For each single feature method that makes up of the proposed LRMR-Ri & W-GDipC method was used as the comparison method. The experimental results show that the proposed LRMR-Ri and W-GDipC method can significantly improve the accuracy of antifreeze proteins prediction compared with these single feature methods. Moreover, the method achieves ACC of 79.83%, 72.57% and 80.15% respectively on membrane protein dataset based on DT, SVM and SGD classification algorithms.

Especially, with antifreeze proteins dataset and SVM classifier, the values of ACC, RE and MCC of LRMR-Ri and W-GDipC have reached as high as 95.56%, 97.06% and 0.9105, respectively, much higher than those of each single method: Lasso, Ridge, Mic and Relief, nearly 13% higher than single Lasso for ACC. With membrane protein dataset and SGD classifier, the values of ACC, RE and MCC of LRMR-Ri and W-GDipC have achieved 80.15%, 44.6% and 0.6562, respectively, also better than other methods, and around 10% higher than each value of Lasso. Therefore, LRMR-Ri is superior to the other four feature selection methods not only in the prediction of antifreeze proteins but also in the prediction of membrane proteins. The proposed method is superior to other machine learning methods in predicting the performance of antifreeze proteins data. The related source codes and dataset are available at https://github.com/Xia-xinnan/W-GDipc-LRMR-Ri. 

In future research, different strategies can be used for the fusion of feature representation of protein sequences, such as a new feature extraction method based on pK value, which represents the dissociation constant of the amino acid, and amino acid frequency [35]. Different optimization algorithms can also be used to optimize the fusion coefficients [36]. For example, the particle swarm optimization algorithm can be used to optimize the parameters [37]. Because particle swarm optimization (PSO) is easy to fall into the local optimal solution, once the dimension increases, its performance will decline in some degree, so better-performing parallel particle swarm optimization (PPSO) can also be introduced and used to find the optimal parameter and so on [38–41].

At last, we want to give an explanation about the choice of using five-fold cross-validation. In this test method, we selected four of them for training and one for testing in turn and the average of the results of five is used as the estimation of the accuracy of the algorithm, which relatively avoids the risk of data leakage. There are three main reasons for not using the independent testing datasets in this paper. First, according to literature [11], and two references which recently cited it, a review article [42] and another one [43], there are quite a few papers which focused on biopeptides, novel therapeutic peptide functions and short antimicrobial peptides only used cross validation for test, which enhances the possibility for our W-GDipC method to compare with them. Second, the segmentation of training, verification and testingdataset sometimes may possibly lead to over-fitting, for there is a great randomness in the segmentation. For example, the independent set test is verified on the test set only once, which leads to the expansion of the randomness, while we can use the cross validation to reduce this randomness by dividing data several times and get a better robustness through the average method. On the other hand, how to reasonably divide training set, verification set and test set is a challenging problem, which is sometimes subjective, thus leading to the risk of insufficient model training or over fitting. Third, since we do not use deep learning algorithms in this paper and thus the number of parameters is not large, it is not so necessary to pay for the cost of time complexity and data segmentation due to independent dataset. Of course, for future study, some constructive approaches such as independent testing datasets method or others can also be attempted.

## Methods

### Dataset

In this study, we used the antifreeze proteins (AFP) dataset as the benchmark dataset 1 in case of binary classification, and the membrane protein dataset [44] as the benchmark dataset 2 in case of multiple classification. The dataset of AFPs contains 480 positive samples and 374 negative samples. The 480 positive samples were confirmed by biological methods, and selected from Kandaswamy et al. [26] and Zhao et al. [27], whose construction steps are as follows. First, we extract the initial AFP sequences from Pfam database [45]. Then we use PSI-Blast with a E-value of 0.001 to search for each sequence in the non-redundant database and only retain the proteins of AFPs [46]. Finally, we use CD-HIT [47] to remove the sequences whose similarities are large than or equal to 40% from the dataset [30]. The 374 negative samples were selected from the cell penetrating peptide sequences in CPPsite 2.0 [48] and independent of AFP. For the benchmark dataset 2, there are eight categories of membrane protein sequences, with a total of 7582 proteins. The specific dataset information is shown in Tables 4 and 5.

**Table 4:** Antifreeze proteins dataset (Binary classification data)

Benchmark dataset 1		
	Sample types	Quantity
1	AFP	480
2	Non-AFP	374
	Total	854

**Table 5:** Membrane protein dataset (multi-classification data)

Benchmark dataset 2		
	Sample types	Quantity
1	Type I	1054
2	Type II	390
3	Type III	30
4	Type IV	56
5	Mutipass	4581
6	Lipid-chain-anchored	189
7	GPI-anchored	228
8	Peripheral	1054
	Total	7582

### Weighted generalized dipeptide composition (W-GDipC)

Dipeptide composition (DipC) is a feature extraction method that two adjacent amino acids are combined and the frequency of occurrence of residues are calculated. There are $20 \times 20$ possible amino acid combinations of feature vectors of the extracted protein sequences [49, 50]. DipC can be defined by Eq. (1) [51]:

1 $$DipC(i) = \frac{{f(i)}}{{n - 1}},\quad i = 1,2, \ldots ,400$$

where $f(i)$ represents the number of the ith dipeptide, $n$ indicates the length of the antifreeze proteins sequences.

The generalized dipeptide composition (GDipC) is not limited to count the frequency of occurrence of adjacent doublets, but counting the frequency of occurrence of residue pairs with isometric intervals [8, 11]. According to [11], the mathematical expression for the residue pairs with isometric intervals in generalized dipeptide composition is:

2 $$gdipc(k) = \{ q_{1} q_{{2 + k}} ,q_{2} q_{{3 + k}} , \ldots ,q_{j} q_{{j + 1 + k}} , \ldots ,q_{{n - 1 - k}} q_{n} \}$$

where $q_{j} q_{{j + 1 + k}} ,1 \le j \le n - 1 - k$ represents a pair of residues consisting of residues with isometric intervals, $k_{{(k \ge 1)}}$ indicates an isometric interval between two pairs of residues [11]. Then we gave the following notation:

3 $$GDipC(k) = \left( {\frac{{f^{'} (1)}}{{n - k - 1}},\frac{{f^{'} (2)}}{{n - k - 1}}, \ldots ,\frac{{f^{'} (400)}}{{n - k - 1}}} \right)$$

where $f^{'} (i)$ is the number of the ith kind of dimers in gdipc(k). When k = 0, GDipC(0) is DipC.

In [11], researchers arranged all GDipC(k), $(0 \le k \le n - 2)$ into a vector with $400 \times (k - 2)$ dimension, whose possible maximum dimensional value can reach $400 \times (n - 2)$. For short sequences of cell-penetrating peptides, the authors in [11] reasonably chose k = 1, 2, 3 to form 800-D, 1200-D and 1600-D vectors, respectively. Then they used LDA method to reduce the dimensionality and redundancy. Generally, for heterogeneous feature data after pattern mapping, data redundancy is inevitable, which will impair the effectiveness and efficiency of data processing [52]. Therefore, a generalized dipeptide composition with a weighted fusion of sequences feature extraction methods (W-GDipC) is proposed in this paper, which is fused by GDipC(k) and DipC with certain weighting coefficient. Since GDipC is more suitable for short sequences, the fusion of GDipC(k) and DipC allows both long and short sequences to get a better feature expression through proper weights.

The equation of W-GDipC is as follows:

4 $$W{\text{-}}GDipC = \sum\limits_{{k = 0}}^{d} {\alpha _{k} } GDipC(k)$$

where $$\sum\nolimits_{{k = 0}}^{d} {\alpha _{k} = 1,} {\text{ }}\alpha _{k} > \alpha _{{k + 1}} ,{\text{ }}d = 1,2, \ldots ,n - 2$$.

In Eq. (4), $\alpha _{k} > \alpha _{{k + 1}}$ implies that the importance of generalized dipeptide decreases with the increase of the interval length of k.

**Fig. 6:**
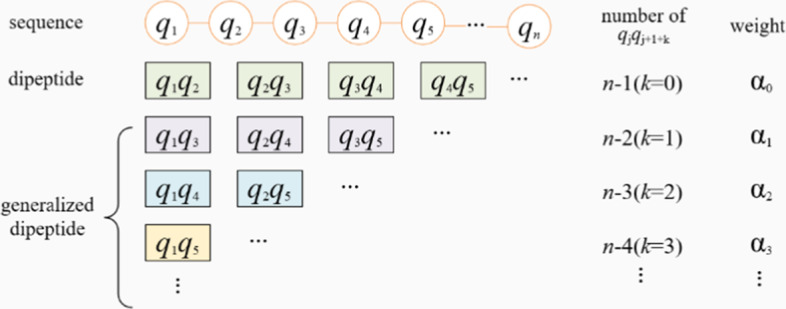
Composition Analysis of W-GDipC

The feature representation of W-GDipC is shown in Fig. 6.

W-GDipC method is proposed based on the characteristics of antifreeze protein sequences. Generalized dipeptide composition in [11] is more suitable for short peptide biological sequences with outstanding specific function than the long AFP sequence here. As a way to eliminate the data sparsity of the traditional dipeptide composition (DipC), W-GDipC not only can preserve the important features of the generalized dipeptide composition algorithm, but also further complete the missing features, which greatly eliminates redundant features and enriches the feature expression of antifreeze protein sequences.

Especially, in this paper, considering the length of AFP sequences comprehensively, we use the following simple expression:

5 $$W{\text{-}}GDipC = \alpha DipC + (1 - \alpha )GDipC(1)$$

where $\alpha$ is the fusion coefficient. In the following section, to simplify the notation, GDipC(1) is denoted as GDipC in the case of no confusion.

There are two advantages of W-GDipC:

1. Data redundancy can be effectively reduced and a clear representation can be obtained after data fusion of DipC and GDipC;
2. The sparseness of local data resulted from singly using GDipC or DipC is avoided to a large extent, so existing feature data is more completely and concisely for long sequences.

Finally, how to select fusion coefficients is also an important step. Specifically, in Eq. (4), how to select $\alpha _{k} ,{\text{ }}d = 1,2, \ldots ,n - 2$, satisfying $\sum\nolimits_{{k = 0}}^{d} {\alpha _{k} = 1,} {\text{ }}\alpha _{k} > \alpha _{{k + 1}}$, so as to get prediction results as good as possible, is a very complex and challenging search process. It is a combination optimization problem with high computational complexity. Commonly used methods for calculating feature weights include Boolean weights, word frequency weights, entropy weights etc. In addition, just like reference [20], when they searched the high dimensional fusion balance factors, they used the genetic algorithm. In [22], when researchers studied the kernel parameter selection method, besides the general grid searching method, they used a clever principle, that is, the optimal kernel parameter makes the reconstruction errors have a reasonable difference between the internal samples and the edge samples. In this paper, since a simplified Eq. (5) is studied, it is relatively easy to find a good α value. In order to find a suitable α, we search it from 0 to 1 with a fixed step in the experiments.

### Ensemble feature selection (LRMR-Ri) method based on two-stage multiple regressions

Inspired by the superimposed generalized learning framework [53], a hierarchical ensemble feature selection method named LRMR-Ri is proposed in this paper, which means Lasso, Ridge, Mic and Relief (abbreviated as LRMR) in the first stage and Ridge (abbreviated as Ri) in the second stage. Specifically speaking, four local optimal feature subsets are generated by using Lasso, Ridge, Maximal information coefficient (Mic) and Relief firstly. If there are some common features in the four feature subsets, the common features could be placed directly into the optimal subset, otherwise, the four feature subsets would be put into the public collection and be selected again by the Ridge filter as at the second stage, which is called two-stage method.

The specific operation of each single filter in LRMR-Ri is as follows.

### Lasso

The characteristic of lasso regression is that it is a generalized linear model with various dependent variables, including one-dimensional continuous dependent variable, multi-dimensional continuous dependent variable, non-negative times dependent variable, binary discrete dependent variable and multivariate discrete dependent variable etc. [54]. In addition, Lasso can also filter variables and reduce the complexity of the model. The variable filtering here refers to not putting all variables into the model for fitting, but selectively putting variables into the model to get better performance parameters.

Lasso has its own independent variable selection function, so the problem here is changed into how to choose the appropriate constant *c* so that *n* independent variables we want can be selected. Since there is a strict positive correlation between the value of *c* and the number of selected independent variables, we use dichotomy to optimize it directly, and give an initial *c* value in 0–1,000,000. The iteration ends with a convergence condition that the number of selected independent variables is greater than or equal to *n* and less than or equal to *n* × 1.05. At the same time, the default value for the maximum number of iterations is 100. If it does not converge after 100 iterations, the initial interval is magnified ten times and the iteration is over again. The process of iterating is repeated up to 10 times. If it doesn’t converge after 10 repetitions, it is defaulted to no convergence. Due to the number of independent variables selected at the final convergence position is not necessarily exact *n* (but must be larger), we select the maximum *n* coefficients among all the independent variables selected by Lasso as the final result.

### Ridge

Ridge regression [55] is the least square regression with Euclidean norm as the penalty. In the least square estimation method, *b* = (*X*′*X*)^−1^*X*′*Y*,where (*X*′*X*) cannot be 0. However, when the correlation between variables is strong, (*X*′*X*) is very small, even tends to 0.

Ridge regression is a biased estimation method for collinear data analysis. By giving up the unbiasedness of the least square method, the regression coefficient obtained at the cost of losing part of the information and reducing the accuracy is more practical and reliable. In essence, a non-negative factor is artificially added to the main diagonal elements of the independent variable information matrix, i.e.,

6 $$b(\lambda ) = (X^{\prime}X + \lambda I)^{{ - 1}} X^{\prime}Y$$

When $\lambda = 0,b(\lambda ) = b$; if $\lambda \to \infty$, $b(\lambda ) \to 0$. The track of $b(\lambda )$ changing with *λ* is called ridge trace. When processing highly correlated data, we usually draw the ridge trace and select an ideal *λ* corresponding to certain stability.

Since the Ridge algorithm itself does not make variable selection, we solve out the optimal regular coefficient through the grid search considering the principle of regularity, then select *n* feature vectors whose absolute value of coefficient is the largest over the optimal regular coefficient as the result of the selection of the independent variables.

### Mic

Maximal Information Coefficient (Mic) [56] is used to measure the linear or nonlinear intensity of the two variables X and Y, and is calculated by the mutual information (MI) and meshing methods. The calculation method is as follows:

7 $$Mic(R) = \mathop {\max }\limits_{{xy < K(m)}} \left\{ {\left. {\frac{{MI^{*} (R,x,y)}}{{\log \min \left\{ {x,y} \right\}}}} \right\}} \right.$$

where $k(m)$ is the size of the grid, which is a variable, usually 0.6 power of the amount of data, R represents the dataset,$MI^{*} (R,x,y)$ is defined as follows:

8 $$MI^{*} (R,x,y) = \max \left( {\sum\limits_{{x \in X}} {\sum\limits_{{y \in Y}} {P(x,y)\log \frac{{P(x,y)}}{{P(x)P(y)}}} } } \right)$$

where $P(x,y)$ represents the joint probability density of variables X and Y, $P(x)$ and $P(y)$ represent the marginal probability density of variables X and Y.

The calculation of Mic is divided into three steps. First, given *i* and *j*, the scatter diagram composed of two variables X and Y is gridded with *i* columns and *j* rows, and the maximum mutual information value is calculated. Second, normalize the maximum mutual information value. Third, select the maximum value of mutual information under different scales as Mic value.

The computational logic of Mic is very simple, which is traversing all feature vectors and calculating the Mic of *y* and it to take the maximum *n* independent variables.

### Relief

The relief algorithm [57] is a feature weighted filtering algorithm, which generates weight according to the correlation between features and classification. If the value of a feature is less than a certain threshold, the feature will be filtered. The relief algorithm is as follows:

Set the train dataset is D, the sampling frequency is m, the number of original features is N, the threshold of feature weight is θ, and the output is the weight T of each feature.

**Table:** 

1. Set the weight of all features is 0 and T is empty set;		
2. for i = 1 to m do		
	2.1. Randomly select a sample R from D;	
	2.2. Find the k-nearest neighbor samples H and M of R from the same sample set and different sample sets of R;	
	2.3. for A = 1 to N do	
		W(A) = W(A)-diff(A,R,H)/m +
	diff(A,R,M)/m;	
3. for A = 1 to N do		
	3.1. if W(A) > = θ	
		Add the A-th feature weight to T;
end		

In 2.3 described by the above algorithm, diff(A, R, H) represents the difference between the samples R and H on the feature A, and its calculation formula is as follows:

9 $${\text{diff}}(A,R,H){\text{ = }}\left\{ {\begin{array}{*{20}l} {\frac{{\left| {R[A] - H[A]} \right|}}{{\max (A) - \min (A)}}\quad {\text{if}}\;{\text{A}}\;{\text{is}}\;{\text{continuous}}} \hfill \\ {0\quad {\text{if}}\;{\text{A}}\;{\text{is}}\;{\text{discrete}}\;{\text{and}}\;R[A] = H[A]} \hfill \\ {{\text{1}}\quad {\text{if}}\;{\text{A}}\;{\text{is}}\;{\text{discrete}}\;{\text{and}}\;R[A] \ne H[A]{\text{ }}} \hfill \\ \end{array} } \right.$$

The W-GDipC and LRMR-Ri methods we proposed are described as follows:

- Step 1: Using DipC to generate $854 \times 400$ and $7582 \times 400$ matrices.
- Step 2: Using GDipC to generate 854 and 7582 400-dimensional feature vectors.
- Step 3: The weighted fusion of the features in step 1 and step 2.
- Step 4: Using Lasso, Ridge, Mic and Relief selecting features to generate 4 feature subsets respectively.
- Step 5: If there is a common feature set among the 4 feature subsets, it is the optimal subset, otherwise it goes to the next step.
- Step 6: Using Ridge for feature selection, the resulting feature subset is the optimal subset.
- Step 7: Put the obtained optimal subset into the classifier.

**Fig. 7:**
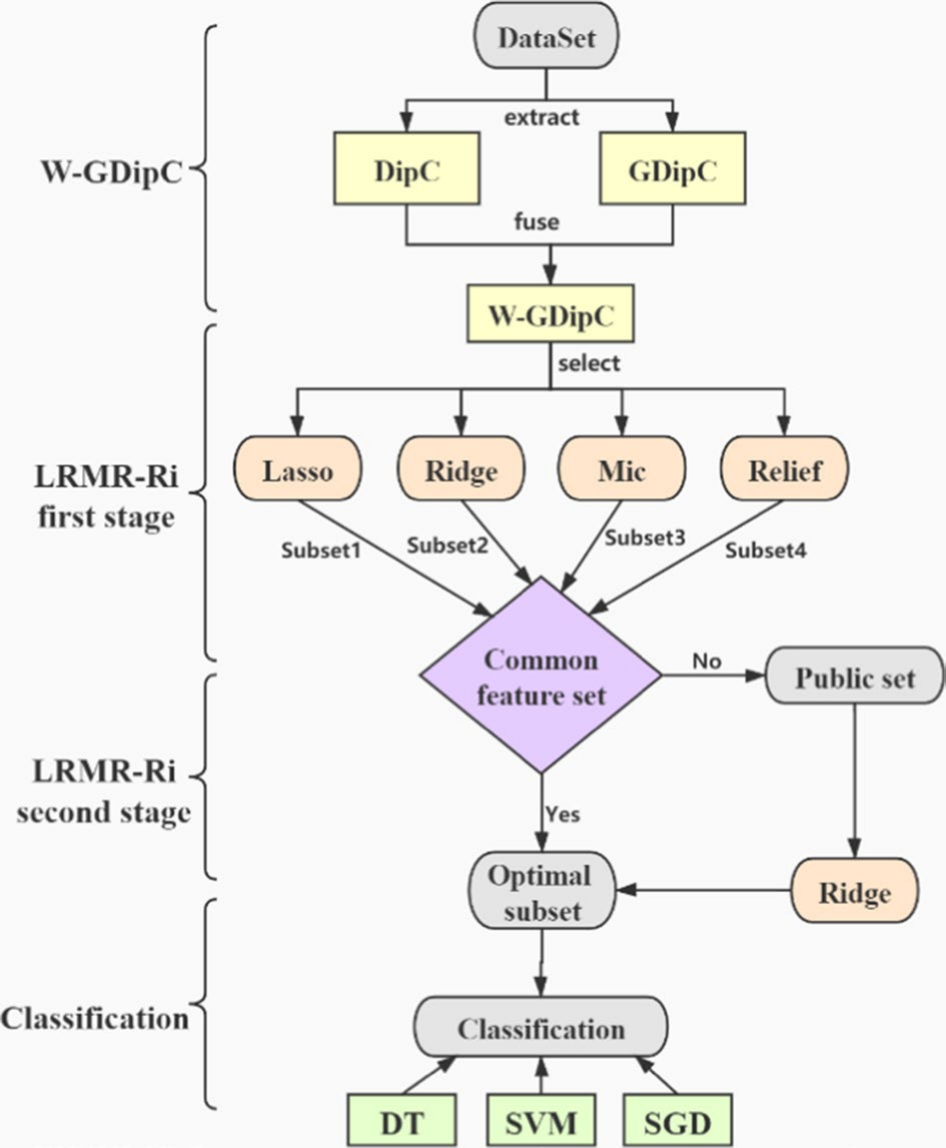
The overall flow chart of W-GDipC and LRMR-Ri methods

The overall flow chart of the W-GDipC and LRMR-Ri methods proposed in this paper is shown in Fig. 7.

### Validation method and performance evaluation index

In statistical prediction, the following three cross-validation methods are commonly used to test the effectiveness of prediction models in practical applications: independent dataset testing, k-fold cross-validation, and jackknife testing. In the prediction studies of antifreeze proteins and membrane proteins, we used the k-fold cross-validation and set k to be 5. Positive and negative samples of the antifreeze proteins and 7582 membrane proteins dataset were randomly divided into five subsets in the five-fold cross-validation. In these five subsets, one of the subsets is retained as a test set, and the remaining four subsets are used as train set. The cross-validation process is then repeated five times with each subset being used as test data in turn. Then average the results of the five predictions as the final output [58, 59].

In this paper, five common evaluation indexes are used to measure the prediction results: accuracy (ACC), recall (RE), precision (PE), F-Measure and Matheus correlation coefficient (MCC). They are defined as follows [60]:

10 $$Accuracy = \frac{{TP + TN}}{{TP + FP + TN + FN}}$$

11 $$Recall = \frac{{TP}}{{TP + FN}}$$

12 $$Precision = \frac{{TP}}{{TP + FP}}$$

13 $$F{\text{-}}Measure = \frac{{2 \times Precision \times Recall}}{{Precision + Recall}}$$

14 $$MCC{\text{ = }}\frac{{TP \times TN - FP \times FN}}{{\sqrt {(TP + FP)(TN + FN)(TP + FN)(TN + FP)} }}$$

Among them, *TP* is the number of correctly recognized antifreeze proteins or category C membrane proteins; *TN* is the number that correctly recognizes non-antifreeze proteins or classification membrane proteins of category C incorrectly into other categories; *FP* is the number of misidentified antifreeze proteins or the correct classification of other membrane proteins categories into category C, and *FN* is the number of the misidentified non-antifreeze proteins or misclassified of other membrane proteins categories into category C. The closer the value of the above index is to 1, the better the performance of the classifier.

## Data Availability

The related source codes and dataset are available at https://github.com/Xia-xinnan/W-GDipc-LRMR-Ri.

## Abbreviations

AFPs: Antifreeze proteins
DipC: Dipeptide composition
GDipC: General dipeptide composition
W-GDipC: Weighted generalized dipeptide composition
Mic: Maximal information coefficient
LRMR-Ri: Ensemble feature selection with Lasso, Ridge, Mic and Relief in the first stage (LRMR) and Ridge in the second stage (Ri)
SVM: Support vector machines
DT: Decision tree
SGD: Stochastic gradient descent
ACC: Accuracy
MCC: Matthew correlation coefficient
PE: Precision
RE: Recall
